# Network-State Modulation of Power-Law Frequency-Scaling in Visual Cortical Neurons

**DOI:** 10.1371/journal.pcbi.1000519

**Published:** 2009-09-25

**Authors:** Sami El Boustani, Olivier Marre, Sébastien Béhuret, Pierre Baudot, Pierre Yger, Thierry Bal, Alain Destexhe, Yves Frégnac

**Affiliations:** Unité de Neurosciences Intégratives et Computationnelles (UNIC), UPR CNRS 2191, Gif-sur-Yvette, France; University College London, United Kingdom

## Abstract

Various types of neural-based signals, such as EEG, local field potentials and intracellular synaptic potentials, integrate multiple sources of activity distributed across large assemblies. They have in common a power-law frequency-scaling structure at high frequencies, but it is still unclear whether this scaling property is dominated by intrinsic neuronal properties or by network activity. The latter case is particularly interesting because if frequency-scaling reflects the network state it could be used to characterize the functional impact of the connectivity. In intracellularly recorded neurons of cat primary visual cortex *in vivo*, the power spectral density of V*_m_* activity displays a power-law structure at high frequencies with a fractional scaling exponent. We show that this exponent is not constant, but depends on the visual statistics used to drive the network. To investigate the determinants of this frequency-scaling, we considered a generic recurrent model of cortex receiving a retinotopically organized external input. Similarly to the *in vivo* case, our *in computo* simulations show that the scaling exponent reflects the correlation level imposed in the input. This systematic dependence was also replicated at the single cell level, by controlling independently, in a parametric way, the strength and the temporal decay of the pairwise correlation between presynaptic inputs. This last model was implemented *in vitro* by imposing the correlation control in artificial presynaptic spike trains through dynamic-clamp techniques. These *in vitro* manipulations induced a modulation of the scaling exponent, similar to that observed *in vivo* and predicted *in computo*. We conclude that the frequency-scaling exponent of the V*_m_* reflects stimulus-driven correlations in the cortical network activity. Therefore, we propose that the scaling exponent could be used to read-out the “effective” connectivity responsible for the dynamical signature of the population signals measured at different integration levels, from Vm to LFP, EEG and fMRI.

## Author Summary

Intracellular recording of neocortical neurons provides an opportunity of characterizing the statistical signature of the synaptic bombardment to which it is submitted. Indeed the membrane potential displays intense fluctuations which reflect the cumulative activity of thousands of input neurons. In sensory cortical areas, this measure could be used to estimate the correlational structure of the external drive. We show that changes in the statistical properties of network activity, namely the local correlation between neurons, can be detected by analyzing the power spectrum density (PSD) of the subthreshold membrane potential. These PSD can be fitted by a power-law function 1/*f^α^* in the upper temporal frequency range. *In vivo* recordings in primary visual cortex show that the *α* exponent varies with the statistics of the sensory input. Most remarkably, the exponent observed in the ongoing activity is indistinguishable from that evoked by natural visual statistics. These results are emulated by models which demonstrate that the exponent *α* is determined by the local level of correlation imposed in the recurrent network activity. Similar relationships are also reproduced in cortical neurons recorded *in vitro* with artificial synaptic inputs by controlling *in computo* the level of correlation in real time.

## Introduction

Assigning a functional role to the correlations in network activity is still controversial. While many studies have proposed that the mean firing rate of the neuron contains much of the information about the sensorimotor interaction with the environment, or the behavioral task being performed [1],[2], other studies have suggested a specific role of higher-order interactions in cortical processing [3]–[5].

Here, we explore another way to extract correlations, through the scaling properties of the power spectrum (hereby called “power spectral density” or PSD) of the membrane potential of single neurons. A particularly common form of frequency scaling is the power-law, according to which the PSD scales as 1/f*^α^* at high frequencies, with some exponent 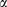 which may be integer or fractional (fractal). Power-law frequency-scaling is ubiquitous in electrophysiological measurements of neuronal population activity, from spiking activity [6] to fMRI signals [7], but its function and origin are still controversial. Some studies consider it as the manifestation of neural “avalanches”, a special form of cell assembly dynamics which would appear when the cortical network is in a critical state [8],[9] and which would be optimal for information processing. Power-law decay functions may also provide the basis for long-lasting interactions in adaptation [10],[11] or memory storage [12].

Several explanations for the origin of power-law scaling have been proposed. At the intracellular level the membrane potential activity was shown to have power-law scaling at high frequencies, with exponent values around 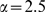 for synaptic background activity *in vivo* [13],[14] and channel noise [15]–[17]. Cable equations predict values of 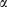 between 3 and 4 for inputs distributed in soma and dendrites, and the non-ideality of the membrane capacitance was proposed to account quantitatively for these values [18]. However, it is unclear whether this exponent can also be modulated by extrinsic factors *in vivo*, and in particular by the synaptic bombardment evoked by sensory input.

As we report in this paper, we decided to approach this issue by analyzing the 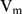 activity of neurons recorded intracellularly in cat primary visual cortex *in vivo*, when the network is driven so as to be in an irregular activity regime. We found that the power-law scaling observed in the intracellular activity PSD at high frequencies is modulated by the stimulus. We examined whether the scaling exponent variations observed *in vivo* can be accounted for by theoretical models *in computo*, using paradigms where the correlation among inputs can be modulated. First, we designed a recurrent network model composed of a thalamic and a cortical layer and showed that when varying the correlation of the thalamic input to the cortical layer power-law exponent modulations were consistent with the *in vivo* results. The scaling exponent thus reflects in this model a specific correlational state of the network imposed by the input. We then dissected out those aspects in the activity impinging on the recorded neuron that can modulate the scaling exponent, and also explored the alternative hypothesis that intrinsic properties of the individual neuron are sufficient to explain the observed modulation. For this purpose, we applied different correlated synaptic inputs to neuron models. This confirmed that a change in the correlation of the synaptic input can modify the power-law exponent. Finally, we investigated this paradigm in cortical neurons *in vitro* using the dynamic-clamp technique and confirmed the results obtained with computational models. We discuss how these results are consistent with the theory that the power-law exponent modulation reflects changes in the correlation state of the network activity.

## Results

### Stimulus Dependence of Frequency Scaling in V1

**Figure 1:**
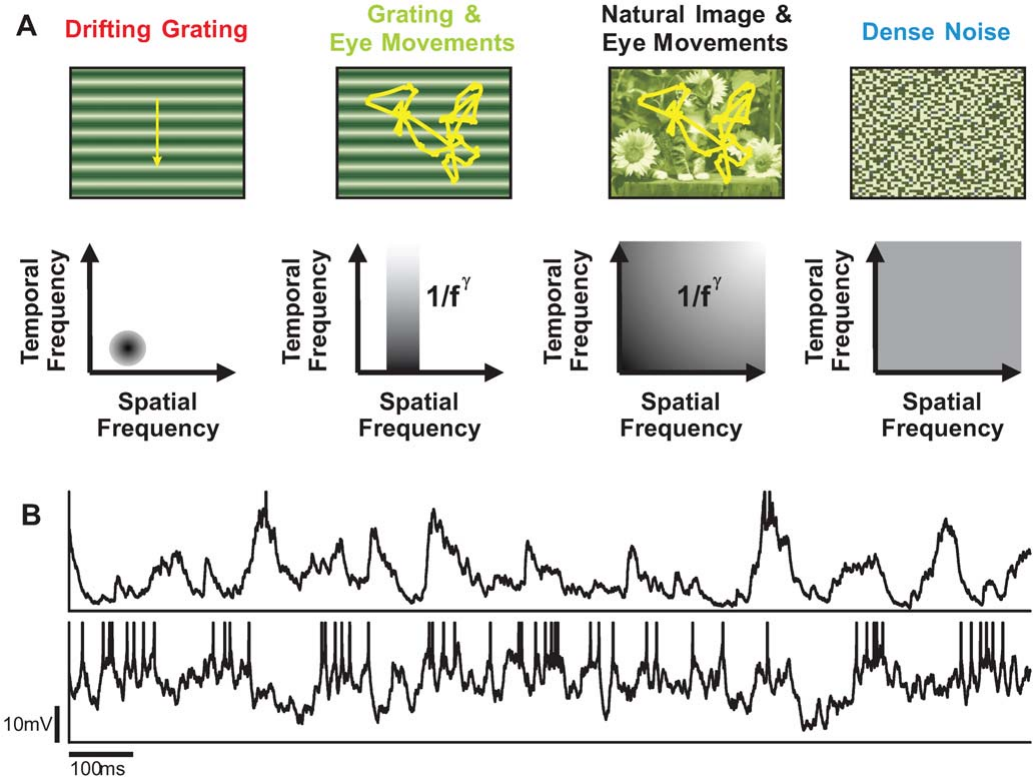
Protocols of visual context dependence. **A:** Stimuli used in the *in vivo* experiments. From left to right: Drifting Grating (DG): a sinusoidal grating with optimal spatial frequency and orientation, drifting at optimal frequency; Grating & Eye Movements (GEM): the same grating animated by a trajectory simulating the dynamics of eye movements; Natural Image & Eye Movements (NI): a natural image animated by the same trajectory mimicking eye movements; Dense Noise (DN): a dense noise of high spatial and temporal definition. All these stimuli were full-field and presented monocularly in the dominant eye. **B:** examples of intracellular responses of the same cell to the NI (top trace) and the DG (bottom trace) stimuli (data from Baudot, Marre, Levy, Monier and Frégnac, submitted; Baudot et al., 2004; Frégnac et al., 2005).

**Figure 2:**
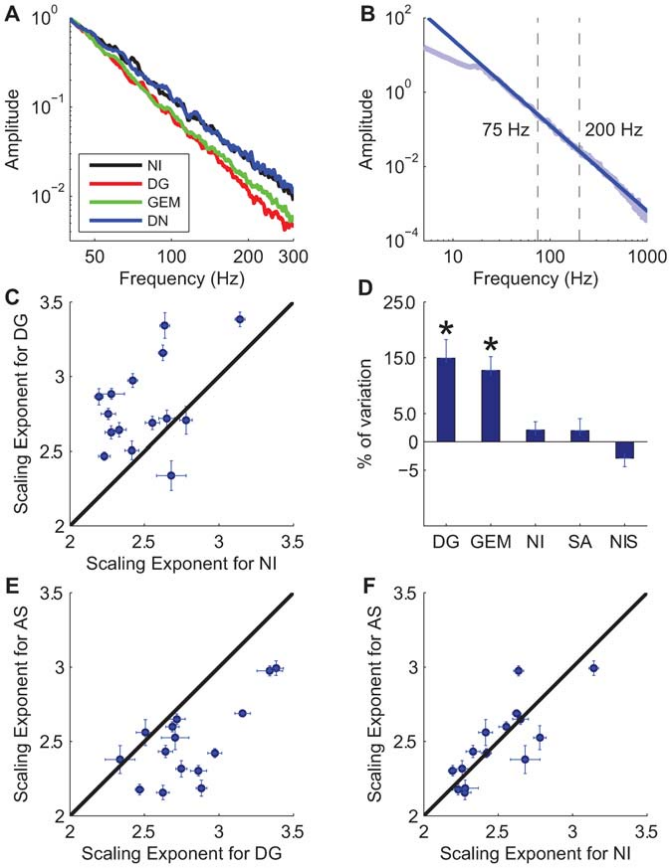
Change of frequency-scaling according to visual context. **A** Power spectral density (PSD) for a given cell in response to the four different stimuli presented in Fig. 1. The traces have been normalized so as to obtain the same value at 40 Hz, for the sake of clarity.**B** Illustration of the linear fit between 75 and 200 Hz for the dense noise protocol. The power-law scaling region extends beyond those frequencies but is affected by synaptic filtering at low frequencies and by noise artefacts at high frequencies. **C** Frequency scaling exponent comparison between DG and NI stimuli for each cell. The error bars represent the standard error of the mean (SEM) on the estimation of the frequency-scaling exponent across the 10 repetitions for each stimulus. The black abscissa line indicates equality between the DG and NI condition. **D** Population analysis relative to the DN case. Each bar indicates the percentage of variation from the DN frequency-scaling exponent. The asterisks (*) indicate a significant difference over the population of cells between the frequency-scaling exponents in response to DN and a given stimulus (paired Wilcoxon test, 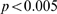). The fourth bar represents the relative change between the spontaneous activity (SA) and the DN condition. **E** Comparison between the frequency-scaling exponent measured during NI stimulation and spontaneous activity (SA) for each cell. The black line indicates equality. **F** Same comparison than **E** between DG and SA.

15 neurons were recorded intracellularly in the primary visual cortex of the anesthetized and paralyzed cat (see Materials and Methods). Each neuron was recorded while presenting four full-field stimuli through the dominant eye (Fig. 1): a drifting grating at the cell's optimal orientation and spatial frequency (DG), a high spatial definition dense noise (DN), a natural image animated with a simulated eye movement sequence (NI), and a grating animated with the same eye movement sequence (GEM). After removing the spikes from the 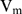 signals by interpolation, we computed their PSDs (see Materials and Methods). These PSDs systematically exhibit a scaling behaviour in a broad, high-frequency band. To extract the scaling exponent, we fitted a linear function to the log-log representation of the PSD, for a range of frequencies from 75 to 200 Hz (Fig. 2B), where the quality of the linear fit is high (mean correlation coefficient 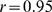). Note that this chosen band is also above the frequencies at which synaptic and membrane filtering cut-off appear [19].

Figure 2A shows the PSDs of the intracellular responses to the four stimuli for the same cell. In the log-log scale representation we observed a dependence of the slope, and hence the frequency-scaling exponent, on the stimulus. To confirm these effects at the population level, we compared for each cell the values of the exponent between pairs of stimuli. Figure 2C shows the comparison between stimuli DG and NI for each cell, and averaged over trials. Although the absolute value of the exponent was highly variable from cell to cell (ranging from 2.0 to 3.5), it was systematically lower, for the same cell, for NI than for DG (paired Wilcoxon test, p
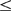
0.003). The magnitude of this difference was much larger than the standard error of the mean (SEM) among the different trials for the same protocol.

We checked whether the value of the exponent could be correlated with the recorded cell's averaged 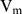 or firing rate. The corresponding correlation coefficients were computed for each stimulus and then averaged together. We found that neither the firing rate (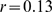) nor the averaged 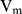 (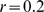) were correlated enough to explain the variations of scaling exponent (although these weak correlations were marginally significant (p
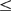
0.07), except for the NI protocol where no correlation was found).

We also estimated whether these systematic modulations were visible at the spiking level, or present only at the 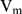 level. We computed the Fano factor exponent (see Materials and Methods) for the *in vivo* spiking responses. In contrast to the frequency-scaling of the 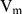, we did not observe any consistent variation of the spiking scaling-exponent with the visual stimulus. Moreover, there was no significant correlation between the 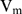 and the spiking scaling exponents (r = 0.2, p
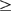
0.1).

In some cells, the same protocol was repeated consecutively, interleaved with 2–3 s of spontaneous activity. We could not see any consistent difference between the power law exponents of the first trial and the others. This means that the dynamics reflected by the power law exponent appear in less than 10 seconds.

These results indicate that the changes of frequency-scaling for the same cell as a function of the stimulus context are mainly determined by the differences in the visual stimulus statistics. Based on the comparison of the frequency-scaling exponents between all possible pairs of stimuli, we divided the stimuli into 2 groups. The exponents obtained from the intracellular responses to DG and GEM were not significantly different from each other but differed significantly from those obtained with NI and DN. We summarized these results by computing the relative changes from DN to the other protocols (Fig. 2D).

For a subset of cells, we also presented three additional stimuli designed as surrogates of the natural stimulus. The “Spatial Random” stimulus is composed of the natural image “scrambled” by randomizing the phases of its Fourier coefficients and animated with the same sequence of eye movements. The “Time Random” stimulus is composed of the natural image animated with a similarly “phase-scrambled” version of the eye movement trajectory. Finally, the “space and time random” stimulus is composed of the scrambled image animated with the scrambled eye movements (plotted as Natural Image Surrogate or NIS in Fig. 2D). These three stimuli evoke power-law exponents similar to the DN protocol (no significant difference, Wilcoxon paired test, p
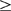
0.32, p
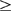
0.014, p
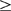
0.13 respectively, and see Fig. 2D for the third surrogate). Even though we did not see a significant difference between NI and DN or between DN and NIS, there is a significant difference between NI and NIS, the latter being the same stimulus with reduced phase coherence (Wilcoxon paired test, p
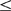
0.003, p
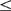
0.003, p
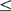
0.006 respectively for the three surrogate stimuli).

From this study, we concluded that the value of the frequency-scaling exponent of the intracellular signal is strongly dependent on the visual input. It is interesting to note that the scaling exponent always seems to be smaller when the stimulus is less correlated (DN being the extreme case where there is no correlation in the stimulus).

### Spontaneous Activity

We applied the frequency-scaling analysis to periods of spontaneous activity recorded in the same cells. Comparison between the frequency-scaling exponent of Spontaneous Activity (SA) and those in response to the five different stimuli was also performed at the population level. We observed a systematic increase from SA to the DG and GEM stimuli (Fig. 2D and Fig. 2F; paired rank Wilcoxon test, p
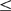
0.0003; the average difference between paired data SA-DG or SA-GEM is significantly different from zero, t-test, p
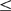
0.0001). In contrast, the SA frequency-scaling exponents are similar to those for DN, NIS and NI (Fig. 2E; for NI r = 0.81, paired rank Wilcoxon test, p
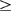
0.5; slope = 0.82; the average difference between paired data SA-NI or SA-DN is not significantly different from zero, t-test, p
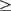
0.1).

### Multifractal Analysis

To estimate how much the frequency-scaling exponent tells us about the multiscale statistics of the intracellular signal, we performed a multifractal analysis (see Materials and Methods). We therefore computed the two first moments of the singularity spectrum over the different cells and protocols.

The first moment is linearly related to the frequency-scaling exponent measured on the PSD [20]. The respective values were indeed correlated over the population. The second moment is slightly above 0 for the four protocols (DG: 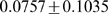, GEM: 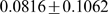, NI: 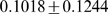, NIS: 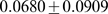 and DN: 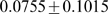), while no significant differences were found between protocols. The intracellular signal is thus very close to a monofractal process, exhibiting self-similar behaviour. Furthermore, the first-order part of the singularity spectrum is the only one which varies with the visual stimulation. The functional sensitivity of our multiscale statistics can be reduced to the power-law behaviour of the 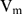 trace.

### Frequency Scaling in a Simple Retinotopic Cortical Model

**Figure 3:**
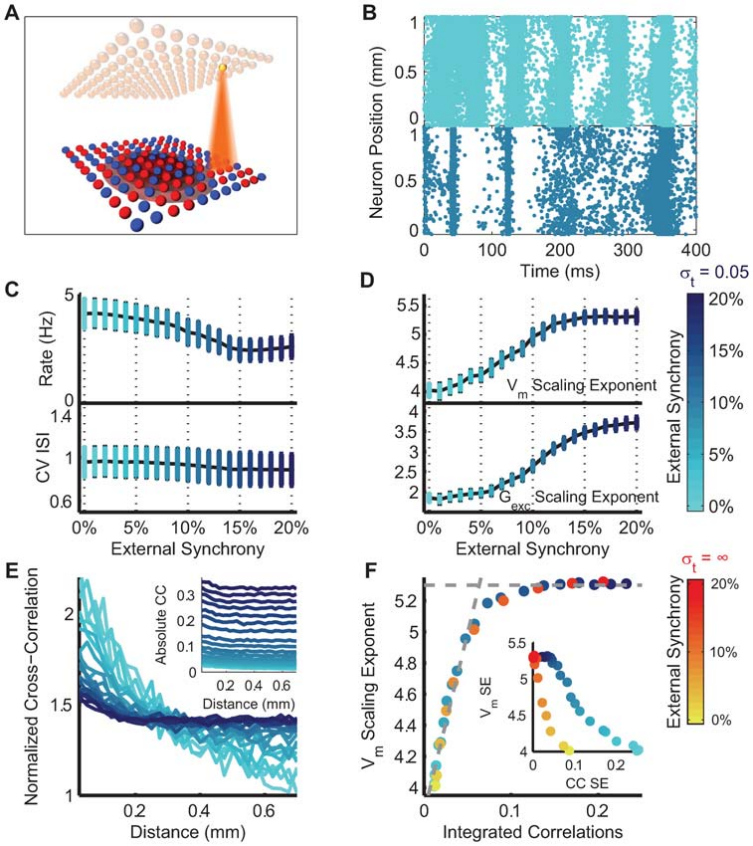
Modulation of the frequency-scaling in a recurrent network model with inputs of variable synchrony and spread. **A** Schematic representation of the network structure and connectivity. The cortical (lower sheet, blue and red neurons) and thalamic input (upper sheet, yellow neurons) layer-like networks (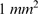) face each other. The cortical neurons are locally connected together, according to a Gaussian distribution (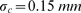) and the retino-thalamic input projects its synaptic connections on the cortical layer through a narrower Gaussian distribution (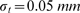). **B** Example of raster plots in the cortical layer in response to two thalamic input synchrony levels (top: synchrony of 0%; bottom: synchrony of 10%). **C** Mean firing rate (top) and coefficient of variation (bottom) of the cortical layer response to thalamic inputs of different synchrony levels. For each simulation, twenty neurons were randomly chosen among the network population to estimate error bars. **D** 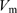 (top) and 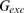 (bottom) frequency-scaling exponents as functions of the input synchrony. Bars indicate standard deviations of the scaling exponent values. **E** Averaged spatial cross-correlation between neuronal activities as a function of the distance between pairs of neurons, for different input synchrony levels, normalized by the total area of the distant-dependent cross-correlation function. Inset: same graph without the normalisation. **F** values of the 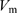 frequency-scaling exponent as a function of the coefficient of correlation integrated over distance. Inset: values of the frequency-scaling exponent as a function of the correlation extent in the network activity (see text). The same results are shown in red for an infinite spread of the thalamic input.

To study the effect of correlated input, we considered a simple model of a cortical network fed by an input with a controlled level of synchrony. This model was shown to be sufficient to reproduce the frequency-scaling exponent modulation measured above. In order to mimic the cortical network and the retinotopy of the input, we simulated topographically-connected networks of excitatory and inhibitory neurons using integrate-and-fire models and conductance-based synapses (see Materials and Methods). We considered networks with topographically organized connectivity where each neuron is connected to its neighbours according to a Gaussian distribution (Fig. 3A).

The stimuli used during *in vivo* experiments have different levels of correlation (Fig. 1A): the DG stimulus is highly correlated across space and time (one Dirac impulse in the spatio-temporal spectral plane), while the DN is, by definition, fully uncorrelated (flat spectrum in space and time). We chose to stimulate the recurrent network model with inputs having different levels of synchrony. The visually driven thalamic inputs project in a local region of space (Fig. 3A), and the cortical response is thus the product of both the thalamic input and the recurrently mediated activity. The different levels of synchrony give rise to responses in the cortical area with different structures (Fig. 3B), although the mean firing rate and the coefficient of variation of the cortical activity remain roughly constant over the different levels of input synchrony (Fig. 3C). In particular, the cortical layer displays spontaneous waves of activity with an irregular and low frequency firing regime (rate
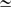
4 Hz and ISI CV
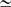
1) when there is no synchrony within the thalamic discharge. The presence of correlation in the external input disrupts these waves and creates synchronous firing in the cortical layer (Fig. 3B).

The frequency-scaling exponent in the model was estimated from the 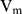 traces of twenty cells (see Materials and Methods). The values of the 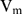 and 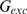 frequency-scaling exponents both increased when the input synchrony increased (Fig. 3D). This also held for the inhibitory conductance 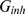 which behaved as its excitatory counterpart (data not shown). This is consistent with the *in vivo* results where stimuli with more correlation (DG, GEM) evoke higher values of the scaling-exponent than the “decorrelated” stimuli (NI, NIS and DN).

### Determinants of the Scaling Exponent

We next examined which features of the network activity structure could be related to this modulation of the scaling exponent. Fig. 3E shows the spatial pairwise cross-correlation between pairs of neuron as a function of the interneuronal distance, for different levels of the input synchrony. The increase in input synchrony resulted in two simultaneous changes: a global increase of the cross-correlation values (Fig. 3E, inset) as well as a flatter spread profile over larger distances; when normalizing by the integral of the correlation over distance, it appears that the fall-off of the cross-correlation function (CC) is steeper for lower levels of synchrony (Fig. 3E). In summary, the different levels of input synchrony modulate not only the global level of the correlation in the cortical network, but also its topographic extent and distance dependence.

We next quantified the two features of the network activity that are modulated by the input synchrony and compared their modulation to that of the 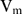 exponent. We first compared the 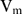 exponent values to the *integrated correlation*, defined as the normalised cross-correlation integrated over distance. The frequency-scaling exponent increased linearly with the integrated correlation (from 0.0 to 0.05) and saturated around 5.25, for an integrated correlation of approximately 0.1 (Fig. 3F).

We also observed that the pairwise correlation between neurons scales with distance when expressed in logarithmic coordinates, which could be related to the 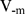 frequency-scaling exponent. The corresponding cross-correlation scaling exponent (CC SE), which reflects the fall-off gradient of the spatial correlation, decreases linearly when the 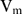 exponent increases (Fig. 3F, inset).

To disentangle the influence of these two factors, we tested the effect of the spread of the thalamic projection to the cortical layer, which parameterizes the extent of the spatial correlation of the inputs. We ran the same simulations with an infinite spread (i.e., the thalamo-cortical connections were random). This condition might be related to the effect of a decorrelated background noise. While the relation between the cross-correlation scaling exponent and the 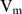 exponent was shifted, the relation between the integrated correlation and the 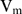 exponent remained unchanged. We found similar results by varying the spread between these two extreme values (data not shown): the spread had no direct influence on the 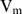 exponent value but shifted the baseline cross-correlation scaling exponent. Thus the variation of the spread, which determines the spatial structure of the input, did not alter the relation between the integrated cross-correlation and the 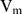 exponent.

This important relationship shows that, in this model, the integrated correlation is detected at the single-cell level through the membrane potential power spectrum scaling property for any stimulus. This measure thus provides a reliable hint about the actual functional state of the network. It also appears that, even if the spatial structure of the correlation is varied, the exponent value remains unchanged. This latter observation could explain why stimuli differing in their spatial structure can produce similar exponents *in vivo*.

As in the previous *in vivo* study, we estimated the Fano Factor scaling exponent. Even when averaging over a population of randomly assigned neurons, the mean Fano Factor did not exhibit any systematic variation with the input synchrony, the integrated correlation or the cross-correlation scaling exponent. This is in accordance with the *in vivo* results.

Finally, it is interesting to note that this network model can reproduce the changes in the frequency-scaling of the 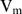 observed *in vivo*, despite its simplicity and the absence of any form of power-law in the spatial rules of connectivity: the thalamo-cortical and the cortico-cortical connectivities are drawn in our simulations from Gaussian distributions. Therefore it is not necessary to implement a scale-free connectivity to observe a frequency-scaling exponent emerging in the synaptic bombardment.

### Frequency-Scaling in Single-Cell Models

We have shown that the 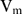 scaling exponent is related to the integrated cross-correlation of the network activity. This integrated correlation depends on at least two factors: the global correlation level of the activity ( *correlation strength*) and the spatial extent of the network correlation (*correlation extent*). In our recurrent network model, both are modified simultaneously when varying the input, which makes the isolation of the precise feature modulating the scaling exponent difficult. We thus turned to the modeling of a single neuron receiving parameterized correlated synaptic noise in order to dissect out the influence of the different parameters of this correlated noise on the postsynaptic 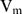 scaling exponent.

Furthermore, although the network model provides a possible explanation for the 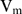 frequency-scaling exponent modulation, this does not exclude a possible alternative hypothesis for our *in vivo* observations : due to the non-linearity in the neuronal transfer function, the 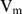 frequency-scaling exponent variation *in vivo* could be due to the variation of the input firing rate or the different depolarisation levels from one protocol to the other.

For these reasons we measured the 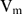 frequency-scaling exponent in isolated neuronal models in response to several correlated synaptic inputs, where all these parameters can be varied independently. We also injected the same correlated synaptic patterns into biological neurons *in vitro* through dynamic clamp. This allowed us to test independently the effect of the correlation strength and extent, and to test the simpler hypothesis aforementioned.

To further understand the relationship between the presynaptic activity and the 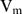 frequency-scaling, we designed a model assuming that the irregular activity originates in the synaptic activity impinging on the recorded cell. Indeed, since the frequency-scaling exponent varies for the same cell and different visual stimuli, it must be linked to the activity of the network surrounding the observed neuron. Note that, being interested only by these relative changes, we did not search for the mechanisms shaping the absolute value of the 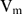 PSD scaling, which may include intrinsic mechanisms [18],[21],[22]. For this reason we show the *relative* modulation of the values of the frequency-scaling exponent in different models and *in vitro* experiments, the baseline being the exponent in response to Poisson stimulation, unless otherwise noted.

In the retinotopic model discussed in the previous section, synchronous input in the thalamic layer evoked synchronous firing in the cortical layer at random positions. These firing assemblies affect the recorded neuron through lateral connections with different propagation delays, which depend on the distance from the presynaptic neuron. The temporal correlations in the presynaptic spike train impinging on the recorded cell thus reflect both the direct thalamic input and the spatial correlations observed in the intracortical distance-dependent cross-correlation. Our aim was to determine how these temporal correlations present in the afferent pattern are conveyed from the presynaptic bombardment to the subthreshold activity through cell integration. Note that the propagation delays play a crucial role in the translation of spatial correlations into temporal correlations. Indeed, if the presynaptic population could interact instantaneously with the postsynaptic cell (no propagation delay), synchronous firing would only increase the membrane potential variance.

**Figure 4:**
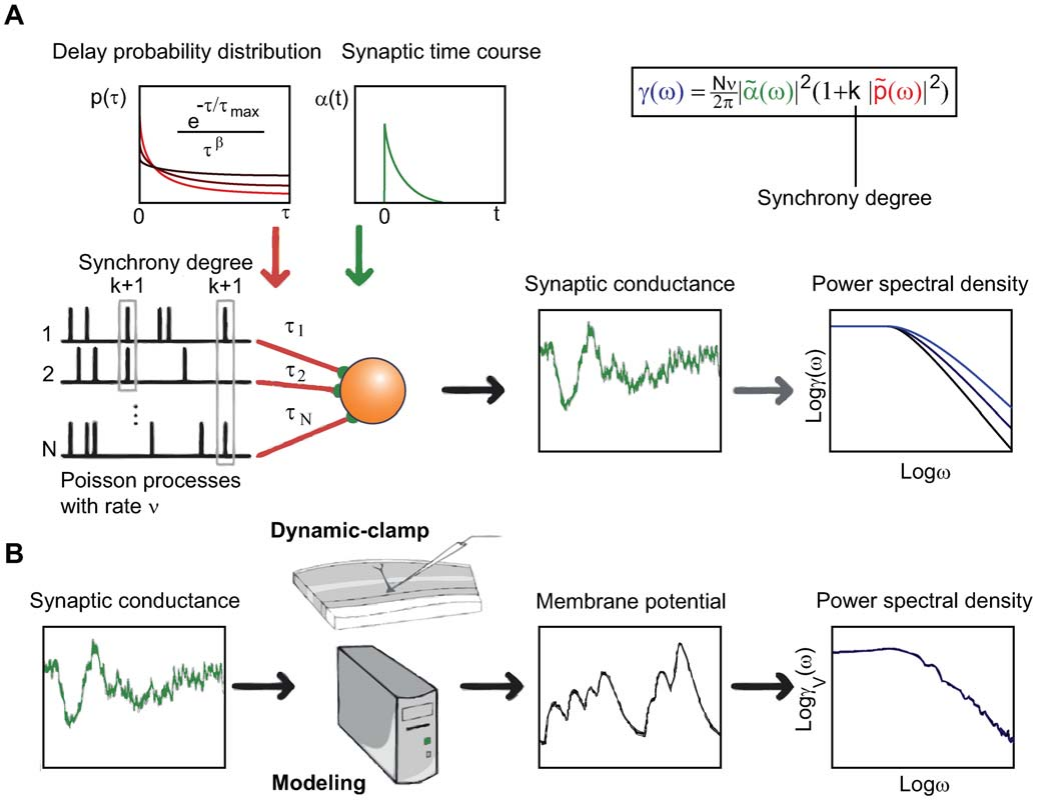
Conceptual scheme of the synchrony generator model and the corresponding conductance injection in model and *in vitro* neurons. **A** Simple representation of the conductance generator. At each time step 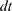, with a probability proportional to the firing rate 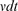, k+1 neurons emit a spike synchronously. These spikes are then conveyed to the postsynaptic neuron with different delays, distributed according to a power-law probability density function (red curves). The arriving spikes then trigger post-synaptic conductances of exponential form (green curve, synaptic time course). The resulting conductance trace 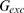 (green trace) has a PSD (blue curve) with a frequency power-law scaling behaviour. The analytical relation between the Fourier transform of the delay distribution and the PSD is given above the graphs. **B** The resulting synaptic conductance is then injected either in a model of single neuron or in a biological neuron through dynamic-clamp (see Materials and Methods). In both cases, the resulting membrane potential is measured and the corresponding PSD is estimated.

The model is composed of 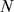 presynaptic neurons (Poisson processes) that all fire at the same mean rate 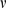, with a constant synchrony fraction 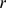. This means that each emission of a spike occurs simultaneously in 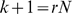 neurons (Fig. 4). These presynaptic neurons then project with different conduction delays to the same postsynaptic neuron, which represents the recorded cell. This means that spikes emitted simultaneously by various presynaptic sources will arrive with different delays at the postsynaptic neuron, thus creating a high-order structured temporal correlation pattern. The delays are chosen randomly according to a distribution 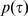 (Fig. 4).

We emphasize that this model is not biologically realistic: it is a correlated spike train generator parameterized by the synchrony level 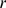 and the delay distribution 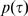. To give more intuition about what these parameters represent, and to make a link with the recurrent model, we can interpret 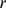 as the strength of the correlations in presynaptic activity, and 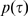 as the way these correlations are temporally distributed. Note that both of these parameters would influence the integrated correlation measured previously in the recurrent model (the spatial correlation in the recurrent model becomes a temporal correlation when considering the delays between distant neurons).

In this model, it can be shown [23],[24] that the analytical expression for the conductance PSD resulting from the synaptic integration of all these inputs is given by Eq. 5
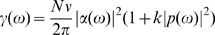
where 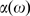 is the Fourier transform of the synaptic time course (when the synapse is exponential, this is a Lorentzian curve), and 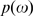 is the Fourier transform of the delay probability distribution.

From this expression, we find that a controlled way to impose an activity-dependent frequency scaling behaviour in this model is to impose a temporal delay distribution having itself a power-law form. Furthermore, this form of correlation is what we found in the recurrent model, although it was not implemented in the connectivity. For this reason the delay distribution will have the form
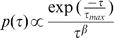
(1)The 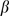 parameter parameterizes the extent of the delay distribution: the higher is 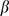, the narrower will be the delay distribution. An infinite value of 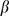 would correspond to all delays equal to 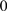. We emphasize that this choice of delay distribution is not *ad hoc*, but rather is imposed in order to control the 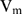 frequency-scaling exponent. Other forms of delay distribution might produce more realistic presynaptic patterns, but we focus here on the part of the correlations that will exert a direct control over the postsynaptic frequency scaling.

The power spectral density of this delay distribution is [6]:
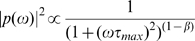
(2)The synaptic conductance 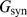 frequency-scaling exponent is thus equal to 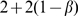 for frequencies beyond the synaptic filtering and the delay cut-offs. Note that, as already shown at the population level in Fig. 3F, the synchrony level detected in the presynaptic train has a “gating” role according to (Equ. 5): no synchrony at all would give a 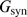 frequency-scaling exponent of 4 whatever the value of 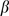. Moreover, the relationship between the exponent and 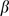 is here uncovered as soon as a minimal level of synchrony is present in the presynaptic population (theoretically, any 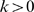).

### Excitatory-Only Simulations

**Figure 5:**
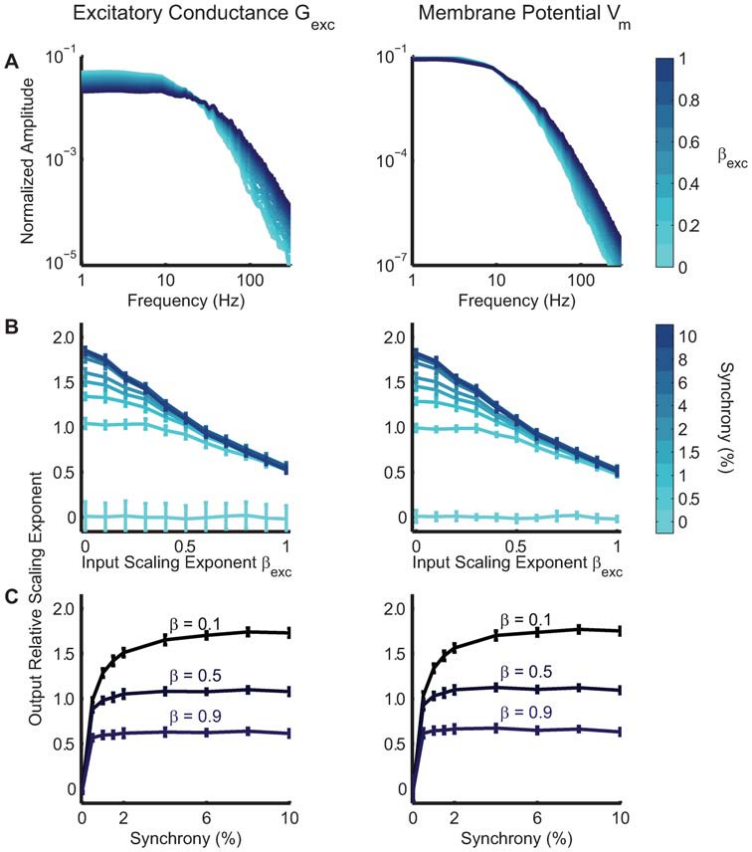
Variation of the value of the frequency-scaling exponent at the conductance and membrane potential levels for excitatory input only as a function of the parameters 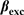 and 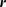 (synchrony percentage). Excitatory conductance 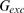 and membrane potential 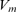 are plotted in the left and right column respectively. **A** Illustration of the PSD modulation on a log-log scale for different values of the parameter 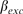 ranging from 0 (light blue) to 1 (dark blue). **B** Variation of the output frequency-scaling exponent with the 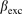 parameter, for different levels of synchrony. When 4% of the presynaptic neurons are synchronous, the relation is almost saturated. **C** The gating effect of synchrony. For three fixed values of 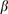 = 0.1, 0.5 and 0.9, the curves represent the modulation of the output frequency-scaling exponent according to percent synchrony.

We numerically simulated this model to check the previous analytical expression. We took a population of 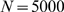 neurons and first fixed the presynaptic firing rate to 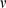 = 10 Hz. For different values of the delay distribution parameter 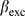, and synchrony 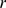, we simulated the model to produce 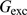 and 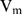 traces. Figure 5A shows the resulting 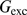 and 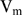 PSDs, for a fixed synchrony level 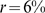, and 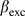 ranging from 0 to 1. The PSD frequency scaling decreases when 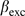 increases for frequencies above 20 Hz.

We then measured the frequency-scaling exponents in these traces to quantify this result (see Materials and Methods) and plotted them as a function of the synchrony level 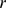 and 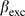 (relative to the Poisson exponent). As predicted, the exponent decreases when the parameter 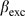 increases (Fig. 5B). This inverse relation between the 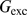 frequency-scaling exponent and 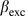 appears more and more clearly as the synchrony 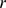 increases, and saturates for 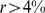 (Fig. 5B). Nevertheless, even with an amount of synchrony as small as 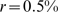, the dependence of the power-law on 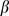 is already monotonic. We obtained a linear relation between 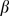 and the output frequency-scaling exponent, although the absolute values are not exactly those predicted by the analytical relation, most probably due to a finite-size bias of the estimation.

To illustrate this “gating” effect of the synchrony, we plotted the frequency-scaling exponent against the synchrony level 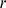, for fixed 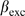 (Fig. 5C). When increasing 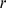, the exponent first increases and then saturates to a plateau which depends on 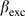.

Identical results were obtained for 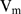 but with a systematic shift of 2 corresponding to the membrane integration (absolute exponent values were between 2 and 4 for the conductance, and between 4 and 6 for 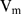). This is what we would expect for a current-based model for which the effect of membrane integration results in a shift of 2 in the frequency-scaling exponent. This shows numerically that the non-linearity induced by the use of conductance-based synapses does not alter this relationship. Therefore, as long as few neuron assemblies are firing simultaneously in the presynaptic population, their correlations are made visible through the postsynaptic membrane potential PSD. Note that the results displayed in panels B and C of Fig. 5 are reminiscent of those obtained for the retinotopic cortical network in Fig. 3F. Indeed, increasing the synchrony or decreasing the 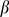 parameter would both increase the integrated cross-correlation, which in turn increases the 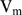 scaling exponent.

### Excitatory-Inhibitory Simulations

**Figure 6:**
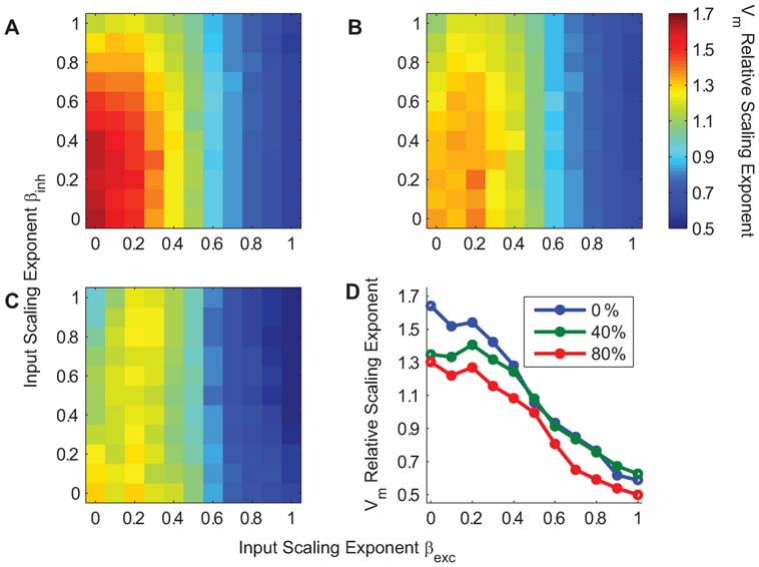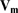 Relative values of the frequency-scaling exponent for different excitatory and inhibitory parameters 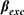 and 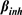. The synchrony percentage 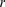 has been fixed to 6% in each simulation. **A** The relative 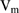 frequency-scaling exponent (color-coded) for 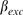 and 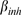 ranging from 0 to 1 without any correlation between excitatory and inhibitory inputs. **B,C** Same graph but with 40% (panel B) and 80% (panel C) correlation between excitatory and inhibitory inputs. In each graph, the excitatory input has a stronger influence on the output frequency-scaling exponent than the inhibitory input. **D** For 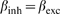, the output frequency-scaling exponent modulation is represented according to different correlation levels.

The synaptic bombardment received by a cortical neuron is composed of both excitatory and inhibitory inputs. We extended our model by adding a population of presynaptic inhibitory neurons which has the same organization as the excitatory population described earlier, parameterized by the synchrony 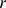 and the delay distribution parameter 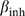. While independently varying the inhibitory and excitatory exponents 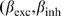, we measured the corresponding 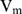 frequency-scaling exponent. We first performed this analysis with the two presynaptic populations having a fixed amount of synchrony (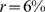), to ensure the impact on the 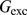 and 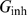 frequency-scaling exponents, and being completely uncorrelated. Fig. 6A shows how the 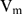 frequency-scaling exponent varies with 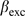 and 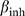. The 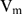 frequency-scaling exponent seemed to be dominated by the 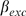 parameter, while the influence of the inhibitory inputs remained marginal. Since the firing rate is similar for excitatory and inhibitory neurons, this dominance was due to the excitatory-inhibitory ratio (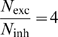). We checked that it was not due to the closer inhibitory reversal potential in additional simulations where we changed the reversal potential (data not shown). Note that when 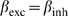, the 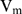 frequency-scaling exponent behaves as in the excitatory-only case (Fig. 6D).

We then examined the case where excitatory and inhibitory inputs are correlated, which is more realistic in view of most of the *in vivo* studies [25]–[27]. The functional relationship between conductance correlations and the 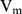 frequency-scaling exponent is conserved for stronger excitatory-inhibitory correlation, although it is slightly affected, especially for small 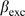 values (Fig. 6B–C). To illustrate this effect, we plotted the variation of the 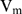 frequency-scaling exponent for 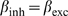 and different levels of correlation (Fig. 6D).

For a sufficient amount of synchrony, the final 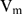 frequency-scaling exponent will thus be mainly influenced by the frequency-scaling exponent of the delay distribution 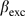, and, to a lesser extent, influenced by the correlation between excitatory and inhibitory conductances, and 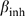. We found that adding a constant delay between the excitation and inhibition as often observed experimentally does not change the 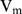 PSD slope value.

To conclude, our model shows how changes in the parameters which determine the correlation in the presynaptic bombardment affect the frequency-scaling exponent of the 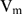 signal. These changes are of the same order of magnitude as that which was observed *in vivo*. Increasing synchrony increases the 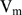 frequency-scaling exponent up to a limit which depends on the 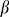 parameters. Increasing 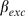 or 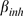, or the correlation between excitation and inhibition, decreases the 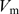 exponent. However, it is much more affected by the correlations present in the excitatory neurons than in the inhibitory ones, since there are many more excitatory neurons.

### Spike and 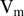 Power Law Relationships

Previous work on power-law frequency-scaling has mainly been based on extracellular recordings, either to characterize single-cell spiking correlations [6] or self-organized avalanche dynamics in networks [8]. Intracellular recordings, as used in the present study, offer a larger sampling of the network dynamics. Indeed, we can ask whether correlations in the synaptic input visible at the 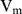 level are still present in the spiking output. We estimated the Fano Factor (FF) for the numerical model to better understand the 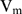-spike frequency-scaling exponent relation.

**Figure 7:**
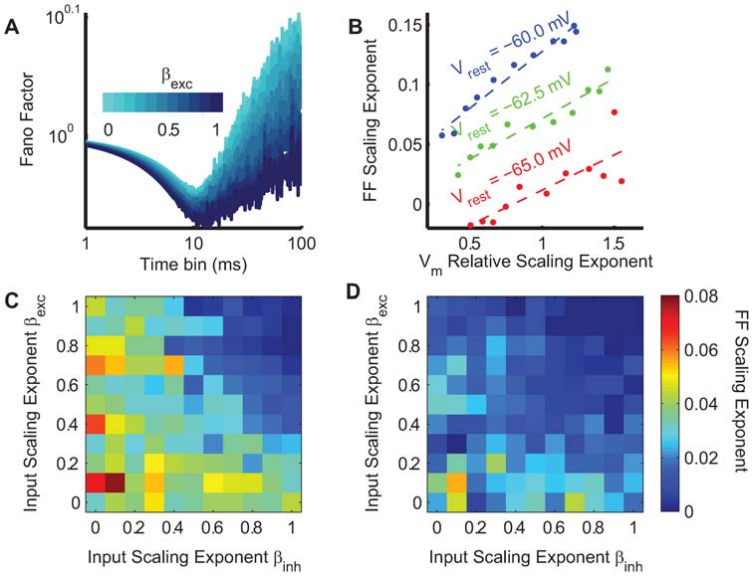
Relation between the 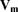 frequency-scaling exponent and that measured from the Fano Factor (FF) of the output spike train. **A** Example of the FF changes as a function of time bin, for different input parameters 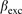. The resting potential 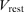 has been set to −60 mV to ensure a large enough number of spikes. The synchrony parameter is fixed at 6%. **B** Relation between spiking and relative 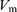 frequency-scaling exponents for different resting potentials (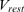 = −65 mV, −62.5 mV and −60 mV). **C,D** Fano Factor frequency-scaling exponents as a bivariate function of excitatory and inhibitory 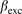 and 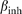 parameters, in the absence of excitatory-inhibitory correlation and for and 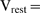 −65 mV (C), and in the case of 40% of correlation and 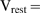 −62.5 mV (D). In this latter case, 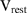 has been increased by a few mV to ensure a reasonable level of spiking activity.

We measured the frequency-scaling exponent in the spiking activity in response to different correlated synaptic input patterns, built by varying the parameters 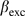 and 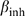. Figure 7A illustrates the Fano factor scaling behaviour for 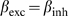 ranging from 0 to 1, and shows a linear increase of the spiking frequency-scaling exponent with 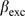 for time bins between 10 and 100 milliseconds. However, we then tested whether the same relationship holds for different resting potentials 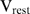 of the postsynaptic neuron (Fig. 7B). It appears that the relation between the spiking and the 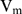 frequency-scaling exponents is strongly dependent on the depolarization level.

This dependency is confirmed when varying 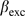 and 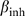 independently. Other parameters can drastically influence the spiking frequency-scaling exponent. As an illustrative example, figure 7C–D show the corresponding spiking frequency-scaling exponents for two different depolarization levels and excitation-inhibition correlation levels; in 7C the postsynaptic 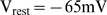 and there is no correlation, whereas in 7D 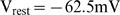 and the correlation is set to 0.4%.

In light of these results, the lack of correlation between 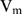 and spiking frequency-scaling exponents, and the absence of systematic modulations for the spiking exponent *in vivo* and in the recurrent model can be explained. This is likely due to the sensitivity of the latter to other parameters that also vary with the stimulus, such as the depolarization level. The spiking frequency-scaling exponent for single-cell study is thus hardly sufficient to characterize the self-similar behaviour of the neural activity. In the *in vivo* data, the FF is measured across a high heterogeneity of depolarization levels, and is thus not reliably linked with the presynaptic correlation. In contrast, the subthreshold activity has shown its robustness to changes in depolarisation, and thus provides a much better insight into the network correlation state, being averaged over a large number of presynaptic spiking neural elements.

### Controls for Different Firing Rates and Resting Potentials

**Figure 8:**
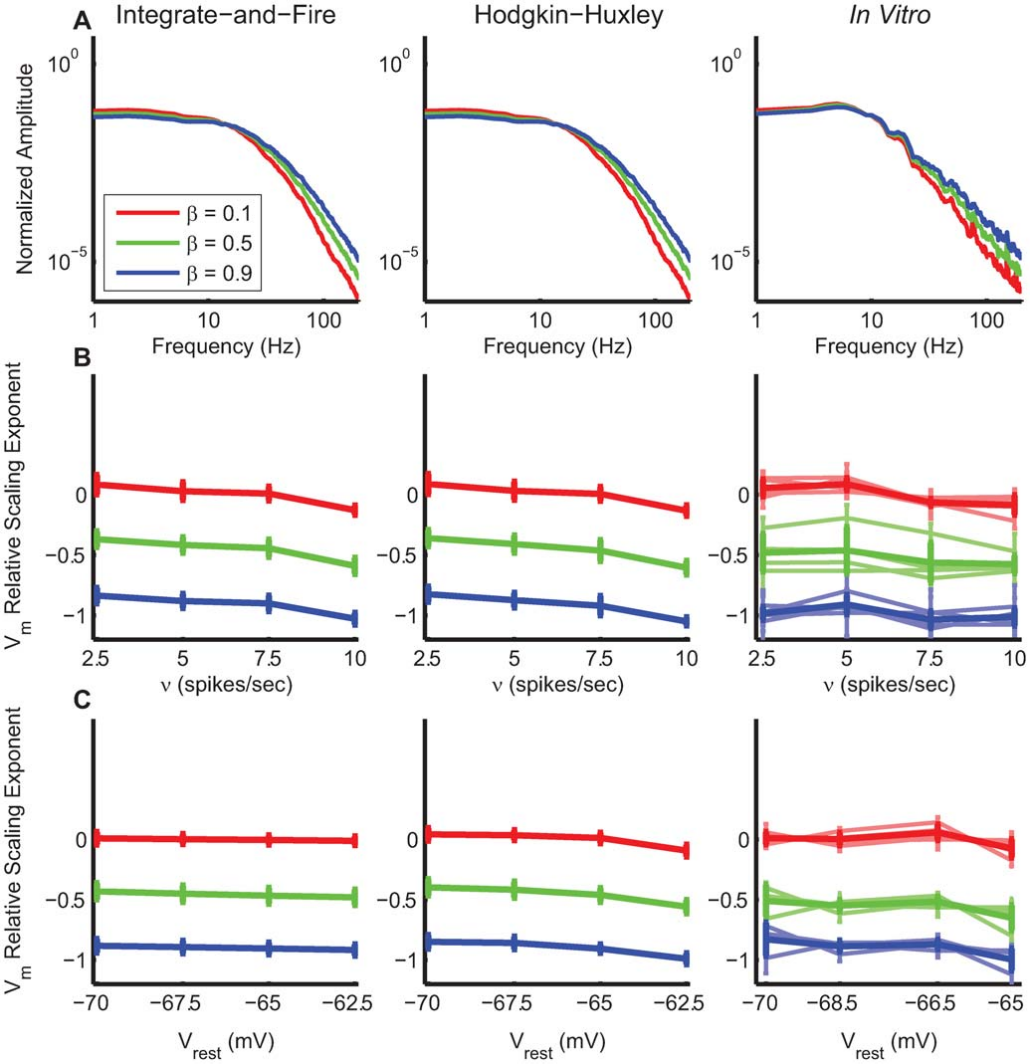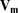 frequency-scaling exponent changes for different input frequencies 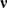 and for different resting membrane potential 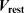. These controls were performed with integrate-and-fire neurons (left column), Hodgkin-Huxley neurons (middle column) and with biological neurons during *in vitro* experiments (right column). The synchrony percentage was kept at 6% and there was no correlation between excitatory and inhibitory synaptic inputs. For the *in vitro* experiments, each light line represents a cell, for which ten trials have been repeated with the same parameters. Error bars are the standard deviation over the trials. The bold line represents the average across cells and trials. Note that the reference value subtracted to each measured exponent is the one obtained when the input parameter 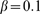 to allow a direct comparison between models and *in vitro* data. **A** PSDs obtained for three values of 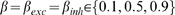. The modulation of the PSD slope is apparent. The absolute slope values are respectively (see Materials and Methods): −3.35 −3.82 and −4.4 (integrate and fire, left); −3.35 −3.82 and −4.4 (Hodgkin-Huxley, middle); −3.28, −3.7 and −3.92 (*in vitro*, right). **B** For three values of 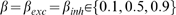, the modulation of the 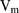 output frequency-scaling exponent according to the mean input firing rate per presynaptic neuron. **C** Same measures according to the postsynaptic resting membrane potential 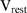.

So far our model has shown how the frequency-scaling exponent can be modulated by the correlations present in the presynaptic activity pattern. However, we had to control for a simpler alternative hypothesis. In *in vivo* data the evoked neuronal mean activity was modulated by the different stimuli (on average 160% decrease from DG to NI), implying that the presynaptic firing rate of the recorded cell varies from one visual stimulus to the other. It is possible that this increase of firing rate induces a change in the frequency power-law scaling. In the following, we call this hypothesis the “first-order hypothesis”. The weak correlation between the cell firing rate and the frequency-scaling exponent observed in the *in vivo* section makes such an hypothesis rather unlikely. However, to directly test this hypothesis on our model, we changed the input mean firing rate from 2.5 Hz to 10 Hz for both excitatory and inhibitory synaptic inputs. For each condition, we computed the 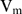 frequency-scaling exponent. Figure 8B (left panel) shows that it is almost unaffected by the input firing rate. Although we observed a small decrease in the frequency-scaling exponent when increasing firing rate, this could still not explain the *in vivo* results. Indeed, in the latter case, even though the correlation is weak, the frequency-scaling exponent increase is concurrent with an increase of the cell firing rate.

We also checked whether the membrane potential level 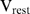 can influence the frequency-scaling exponent. To do so, we varied the recorded cell membrane potential level by adjusting the synaptic strengths (see Materials and Methods). As for the firing rate, no significant influence in the frequency-scaling exponent can be attributed to the depolarization level (Fig. 8C, left panel), confirming the weak correlation observed *in vivo*.

Despite the lack of evidence for the “first-order hypothesis”, our model does not incorporate biologically realisitic integrative features. It has been shown in previous studies [15],[21],[22] that the cell's intrinsic properties, shaped by its ionic channels, could have an impact on the 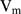 PSD form when the cell is submitted to noisy inputs. We performed the same analysis by replacing the integrate-and-fire model with a Hodgkin-Huxley model. The 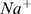 and 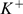 ionic channels could have an influence on the variation of the frequency-scaling exponent. However, adding these mechanisms did not alter the 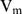 frequency-scaling exponent's dependence on the input firing rate, nor on the mean postsynaptic membrane potential (Fig. 8B–C, middle panel). The results are identical to those obtained with the integrate-and-fire model. Controls were also performed with normally distributed synaptic weights for various standard deviations and gave identical results (Fig. S1A–B). On another set of controls, we changed the synaptic waveform by using synapses with a rise time on the order of 1 ms (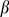-synapse). The controls with this new type of synapse gave identical results to previous cases (Fig. S1C–D).

Apart from the intrinsic mechanisms present in the somatic membrane, a possible source of modulation of the absolute value of the frequency-scaling exponent is the integrative property of the dendritic tree. To test how the dendritic arborization might impact the somatic subthreshold activity, we simulated synaptic input distributed in the dendrites of reconstructed pyramidal neurons. As shown in Table S1, the relative modulations of the exponent are well captured by correlation changes in the model, while global conductance changes had a negligible effect. However, it is important to note that these simulations were done using standard simulation tools (NEURON in this case), and thus used the standard cable equations. It has previously been shown that the standard cable equations cannot reproduce the correct frequency-scaling of the 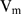 PSD, and that taking into account the non-ideal character of the membrane capacitance could yield the correct frequency-scaling [18]. This could explain why the *in vivo absolute* values of the scaling exponent are not well reproduced here. However, the *relative* modulations of the exponent are well captured by correlation changes in the model, while global conductance changes had a negligible effect.

### Dynamic-clamp experiments *in vitro*

Numerical simulations gave important insights about the role of intrinsic properties in the effects we see, but no computational model can guarantee an exhaustive exploration of such mechanisms. Indeed, even though the first-order hypothesis was invalidated for Hodgkin-Huxley models, we cannot exclude the influence of other ionic currents. Therefore, we performed the same test on real biological neurons through dynamic-clamp *in vitro*.

The correlated conductance traces generated by our model were directly injected into rat visual cortex neurons recorded *in vitro* (n = 9) using the dynamic-clamp technique (see Materials and Methods and Fig. 4B). We performed the same control as above changing the mean input firing rate. The frequency-scaling exponent barely changed (Fig. 8B, right panel; 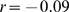, p
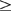
0.3), confirming that the overall presynaptic activity level has a negligible effect compared to the conductance correlations (characterized by the 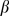 parameter). Even the weak correlation observed between the mean input firing rate and the frequency-scaling exponent has the opposite sign to what is observed *in vivo*. The relative variation for different 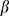 has the same magnitude as the numerical models (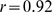).

The previous results were obtained for different resting membrane potentials and did not show any noticeable effect regarding the mean depolarization (Fig. 8B, right panel, 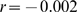, p
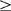
0.9).

In order to measure the influence of the depolarization level on the frequency-scaling exponent, we systematically varied the conductance strength to change the mean 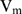 of the recorded cell. The frequency-scaling exponent did not exhibit significant variation (Fig. 8C, right panel). *In vitro* experiments thus confirm our previously observed results from numerical models.

In summary, the correlation in the activity impinging on the recorded cell plays a major role in determining the frequency-scaling exponent of the 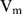. Other parameters, such as the total conductance (see also Fig. S3) and the balance between excitatory and inhibitory conductances, have negligible effects. These results support the idea that changes in the frequency-scaling exponent observed *in vivo* reflect changes in correlations in the external stimulus-driven activity.

## Discussion

In this paper we have analyzed the factors affecting power-law frequency-scaling in the membrane potential of cortical neurons. Our main findings are that (1) intracellular recordings of cat primary visual cortex neurons *in vivo* display power-law frequency-scaling at high frequencies, with a fractional exponent which depends on the spatio-temporal statistics of the visual stimuli; (2) this effect was reproduced in computational models of a recurrent network, and of single neurons of various degrees of complexity; the main determinant of the exponent was the correlation waveform in the presynaptic activity correlation; (3) other factors such as the conductance state had no effect on this measure. These findings were confirmed in cortical neurons *in vitro* using dynamic-clamp injection of random synaptic conductances with controlled degrees of correlation. We discuss below the implications of these findings and how they relate to previous work.

### Influence of Network Correlations and Intrinsic Properties

Our central finding *in vivo* is that the frequency-scaling exponent in V1 is modulated by the visual stimulus statistics. Because such changes are detected in the same cells, they must necessarily reflect changes in the spatio-temporal structure of presynaptic activity. Guided by the fact that intracellular activity in sensory and prefontal cortex shows long lasting temporal correlations, we hypothesized that the main factor affecting frequency-scaling exponents is the correlation in presynaptic activity. This hypothesis was supported by numerical simulations. A similar modulation of the 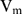 frequency-scaling exponent was also found in a recurrent network for which the input correlation was varied : the scaling exponent increased when the input correlation increased above a certain threshold (required to be detectable). This threshold was not reached during decorrelated states, such as those imposed by surrogate natural scenes.

In the recurrent model, the input correlation modulated both the the absolute strength and temporal structure of correlations. To investigate separate modulations of these two factors, we chose a model of presynaptic inputs with a temporal power-law structure. This choice was made for two reasons: first, because this temporal structure was observed in our network model, without implementing any scaling in the connectivity; second, because it provided an operational way to control the form of the correlations in the input, and isolate which factors influence the output frequency-scaling exponent. The input is thus characterized by its frequency-scaling exponent, and we found that the 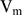 frequency-scaling exponent of the subthreshold output is linearly related to this input exponent. However, this relationship is present only if the correlation strength is large enough. According to these results, the 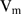 frequency-scaling exponent increase observed *in vivo* could plausibly be due to a global correlation strengthening in the surrounding network and/or by a narrowing of the spatial spread of correlation.

The hypothesis for a determinant role of correlations is also consistent with *in vitro* experiments, where we recreated artificial and controllable synaptic activity by dynamic-clamp. The fact that correlation changes are reflected by changes in the frequency-scaling exponent of the 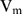 frequency-scaling means that intrinsic cellular properties do not have major dynamical influences on this scaling, and that it mostly reflects synaptic activity controlled by the visual stimulation context. In particular, we showed that neither the mean level of synaptic bombardement nor the postsynaptic depolarization level could significantly modulate the 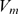 frequency-scaling exponent, even though the cell integrative properties shape its static absolute value [15]–[17],[21].

### A Signature of Avalanche Dynamics?

The finding that 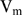 activity presents power-law frequency-scaling is reminiscent of the power-law relationships of self-organized critical states, similar to those found from multi-site local-field potential recordings *in vitro* [8],[28]. In the latter case, self-organized critical states are characterized by the production of “avalanches” of activity, whose size distribution follows a power-law. However, the power-law relations were found there in the frequency domain, which is very different from the distribution of event sizes detected in our study, so our results should not be taken as evidence for avalanche dynamics. We have performed an avalanche analysis on the recurrent network model, and as was reported in a previous study [29], we did not find evidence for avalanche type dynamics in the network during AI states.

Moreover, it has to be noted that the power-law relations found here depend on the stimulus, which means that the frequency-scaling exponent does not represent a unique signature of cortical network activity, but rather reflects a measure of the dynamic interplay between the sensory evoked activity and the ongoing recurrent network activity.

### Relationship between the Subthreshold and Spiking Frequency-Scaling Exponents

Power-law frequency-scaling was reported previously in extracellularly-recorded spiking activity [6],[30],[31]. We observed that the 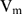 and spiking frequency-scaling exponents are linearly related. However, the exact value of the frequency-scaling of spiking activity critically depends on the 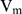 depolarisation level, and thus does not reliably reflect network correlation state. Our study shows that the 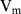 frequency-scaling exponent, which reflects the integration of thousands of input sources, can uncover features of the population activity that were not visible at the single cell spiking level or when assigning a limited number of cells at random.

### Correlation States in Evoked and Spontaneous Activities

Our results imply that tracking the relative changes of the 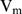 frequency-scaling exponent could be a way to characterize dynamic changes in the correlations hidden in the global connectivity network, but read out at the subthreshold level by each member cell of these overlaid functional assemblies. Having interpreted the relative variations of the frequency-scaling exponent, we can now link these variations with the type of visual stimulus presented.

In order to emphasize the role of dynamic cortical non-linearities in the stimulus-dependency of the power-scaling, we checked whether or not these exponent changes were already apparent in the linear prediction of the 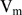 responses. To do so, we used the first-order kernel of the receptive field obtained by dense noise mapping to reconstruct linear predictions of the subthreshold dynamics for the different classes of stimuli and tested the contextual dependency of the spectral scaling properties of the linear predictor. The modulatory effects were not retrieved, which was expected since the estimation of the frequency-scaling exponent is performed on high frequencies (between 75 Hz and 200 Hz) that are not accounted for by the linear kernel (data not shown). We conclude that the exponent variations are not a linear read-out of the scaling behaviour of the stimulus but rather the product of the non-linearities in the input-output relationship imposed by the cortical network.

According to our recurrent network study, the frequency-scaling exponent decreases when switching from DG stimuli to NI or DN stimuli should correspond to a decrease in the correlation strength. Following this interpretation, it could appear surprising that stimuli with very different structures, such as NI and DN stimuli, evoke similar values of the V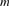 scaling exponent. However, our study showed that the V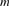 scaling exponent is invariant to changes in the spatial structure of the input. As a consequence, stimuli with different spatial structures can evoke similar scaling exponents provided their global correlation levels are all low.

On the one hand, although it has not been demonstrated directly, natural movie stimuli probably induce decorrelation, for several reasons. First, our natural image is animated most of the time by fixational eye movements, which may already decorrelate activity at the LGN [32]. Second, the decorrelation theory [33] predicts that cortical responses to natural scenes should be decorrelated in order to maximize the transmitted information, and this prediction has been confirmed in V1 studies [34]. On the other hand, dense noise, as a fully uncorrelated stimulus, also evokes a very decorrelated response.

These low correlation levels for both stimuli are probably what make them indistinguishable from the perspective of the scaling exponent. In short, even if the structures of these inputs are very different, thalamic and cortical processing may reduce the initial correlations down to a similar level. Furthermore the scaling exponent captures neither the difference in the spatial structure of these resulting activities nor the difference in the low frequency band dominated by the stimulus spectrum. Taken together these arguments can explain why we observed similar scaling exponents. The same remark holds for DG and GEM stimuli: despite their difference in temporal structure, they might evoke similar levels of correlation, and thus similar scaling exponents, despite the difference in input spatial structure and low frequency content.

Finally, the same argument may explain why we found similar exponents for the spontaneous activity and the natural stimulus: for high frequencies, both exponents likely correspond to a very decorrelated activity, even if there might be a residual synchrony. Note however that this striking correlation between NI and AS is not necessarly present at lower frequencies.

Several studies have compared the structure of the spontaneous activity to that of the evoked activity. The spatial structure of the spontaneous activity measured with voltage-sensitive dye (VSD) imaging has been found to be similar to the DG-evoked activity [35],[36], although this result could not be replicated in awake animals [37]. On the other hand, [38] found that the temporal correlations measured in multi-unit recordings seems to be similar for dense noise, natural scenes and spontaneous activity. Our results and a recent theoretical study [39] seem to be compatible with the latter observations. However, they are not necessarily in total contradiction with the VSD results since our measures concern different frequency bands: while we measured frequency-scaling exponents between 75 and 200 Hz, the VSD measures mostly concerned dye signal fluctuations at frequencies below 20 Hz. It thus appears most likely that V1 responses to natural scenes and spontaneous activity share similar correlation features in the high-frequency band.

We have shown that the frequency-scaling exponents measured in the intracellular activity can vary under the influence of the visual context for the same cell. Our model relates this modulation to a dynamic change in the network correlation state and could be associated to the underlying dynamic dimensionality [40]. Further studies need to address at the population level (LFP or VSD) how the frequency-scaling exponents of the network activity may vary with the stimulus context [41], and if such changes could be indicative of the detection of specific sensory statistics in the external drive or their spontaneous recall by the recurrent structure of the network.

## Materials and Methods

### Animal Experimentation

All *in vitro* and *in vivo* research procedures concerning the experimental animals and their care adhered to the American Physiological Society's Guiding Principles in the Care and Use of Animals, to European Council Directive 86/609/EEC and to European Treaties Series 123 and were also approved by the regional ethics committee “Ile-de-France Sud” (Certificate 05-003).

### *In vivo* Preparation

Cells in the primary visual cortex of anaesthetized (Althesin) and paralyzed adult cats were recorded *in vivo* using sharp electrode (potassium methylsulfate 3 M, 70–100 M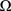) recordings (average 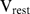 = −67 mV, 0 nA) as described elsewhere [25],[42]. Data processing and visual stimulation protocols used in-house software (G. Sadoc, Elphy, CNRS-UNIC).

### Visual Stimulation

The analyzed data come from *in vivo* experiments to be presented in full in a companion paper (Baudot, Marre, Levy, Monier and Frégnac, submitted). Preliminary accounts have been given elsewhere [43],[44]. Stimuli were displayed on a 21” CRT monitor with a 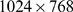 pixel resolution and a 150 Hz refresh rate, with a background luminance of 12 cd/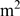. Receptive fields were mapped using sparse noise and classical tunings were determined by automated exploration. Intracellular responses were compared for four full-field visual stimuli of 10 s duration and increasing complexity (see Fig. 1): a) a drifting grating of optimal orientation, direction, and spatial and temporal frequencies (DG), b) the same optimal grating animated by a modeled eye-movement sequence (GEM), c) a natural image animated by the same virtual scanpath (NI), and d) dense binary white noise (DN). The mean luminance and contrast of each movie were equalized. Each movie was presented 10 times. For the NI condition, we used a high definition natural image (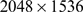 pixels) animated with a virtual eye movement sequence [43],[44] (note that the size of the image is larger than the size of the screen, so that no blank region appears when the image is moved along the oculomotor trajectory). White noise consisted of a dynamic sequence (13.3 ms refresh period) of high spatial definition (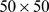 pixels of side length 0.39°) binary dense noise.

### Numerical Models

All the simulations (including dynamic-clamp experiments) were performed with the NEURON software [http://www.neuron.yale.edu] except for the recurrent model which was been run under NEST [45] using the PyNN interface [http://neuralensemble.org/PyNN]. A time step of 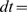
0.1 ms was used systematically. We ran some simulations with 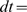
0.01 ms to verify that our results were not dependent on the integration time step (data not shown).

The postsynaptic neuron follows an integrate-and-fire equation with conductance-based synapses whose time evolution is given by
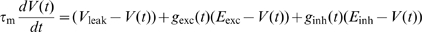
(3)with the resting membrane time constant 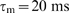, the leak membrane potential 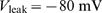 and the excitatory and inhibitory conductances given in units of leak conductance 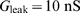. When 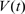 reaches the spiking threshold 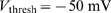, a spike is generated and the membrane potential is reset to 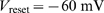 for a refractory period of duration 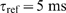. 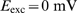 and 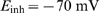 are the reversal potentials for the excitatory and inhibitory exponential synapses 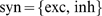 whose dynamics follow
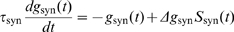
(4)where 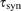 is the synaptic time constant with 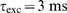 and 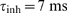. 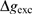 and 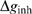 are the quantal synaptic strengths elicited by each presynaptic spike and 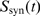 is the point process modelling the incoming spike train. 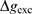 and 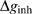 are chosen in order to satisfy the ratio 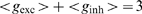 where the brackets signify an average according to 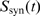, and so that the effective resting potential is 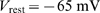 on average. Identical results were been obtained for synapses with a finite rise time (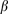-synapses). Parameters for the Hodgkin-Huxley model were taken from [46].

The recurrent network is composed of 10000 excitatory and 2500 inhibitory neurons, sparsely connected, with a connection probability of 2% within each population and between the two populations. The synaptic weights are 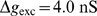 and 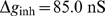. Each neuron has a topographic position on a cortical layer-like surface of 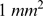, and connects to its neighbours according to a Gaussian distribution of standard deviation 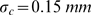. Periodic boundary conditions are used. Conduction delays 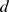 are distant-dependent with 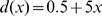 (ms) where 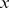 is the distance between the two neurons expressed in millimetres. The slope value of 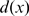 (giving a propagation speed of 0.2 mm/ms) is taken from a previous *in vivo* study showing a lateral propagation speed ranging dominantly between 0.1 and 0.3 mm/ms [42]. The retinotopic drive was modelled as another thalamic layer-like network facing the previous one where each neuron acts as a Poisson process with a controlled amount of synchrony between the firing. To mimic a retinotopic mapping, each cell in the thalamic layer projects to the recurrent network in a topographically organized manner following a Gaussian distribution of standard deviation 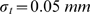 (Fig. 3). The connection probability from the thalamic layer to the cortical layer is also 2%.

In some simulations, we used models based on morphologically-reconstructed neurons from cat cortex, obtained from two published reference studies (layer II–III of cat primary visual cortex Douglas et al. [47]; layer VI of cat somatosensory cortex Contreras et al. [48]), where biological details were given. The three-dimensional morphology of the reconstructed neurons was incorporated into the NEURON simulation environment, which enables simulating cable equations in complex three-dimensional structures [49]. *In vivo*-like activity was simulated in passive models using a previously published model of synaptic bombardment at excitatory and inhibitory synapses [50] (see this paper for details about the parameters and numerical simulations). The density of synapses was constant per unit membrane area according to published morphological studies, and was (per 100 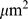): 60 for dendritic AMPA synapses, 10 for dendritic GABA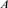 and 20 for somatic GABA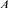 synapses. This gives 9947 AMPA and 2461 GABA*_A_* synapses for the layer II–III cell, and 16563 and 3376, respectively, for the layer VI cell. The release rates, chosen to yield synaptic bombardment consistent with *in vivo* measurements, were 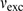 = 1 Hz and 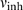 = 5.5 Hz for AMPA and GABAergic synapses, respectively (see details in [50]).

### Correlation Generator

In order to produce spike trains with arbitrary temporal correlations, we used the theory of cluster point processes [23],[51]. The presynaptic activity can be characterized by two main features: on the one hand, the specific temporal structure given by the spike train temporal auto-correlation form, and on the other hand, the correlation strength which measures the temporal coherence between individual presynaptic spike trains (see [52] for a similar distinction). These two features can be controlled separately in the spike train generator composed of a population of presynaptic neurons following Poisson processes, and firing together with a certain amount of synchrony. They project to the postsynaptic neuron through different time delays, randomly chosen from a specific distribution (Fig. 4). The temporal structure is given by the delay distribution whereas the global synchrony in the presynaptic neuronal discharge gives the correlation strength. In our implementation, the presynaptic population is assumed to contain 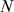 neurons (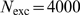 for the excitatory population and 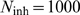 for the inhibitory population, except stated otherwise); at each time step it was decided randomly whether or not some neurons will fire. The probability was adjusted to give a mean firing rate 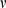 of the inputs. If so, 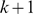 neurons were chosen randomly to fire among the 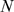 constituting the population. This method allows to have always 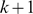 synchronous neurons, and still an apparent Poisson discharge at rate 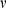 for each presynaptic neuron taken individually. Note that this gives back independent Poisson spike trains when 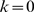. Correlation between excitatory and inhibitory neurons is implemented in the same manner. The delays are then attributed to each presynaptic spike train according to the chosen delay distribution.

From point process theory, this can be seen as two nested point processes. The first point process follows a Poisson process which determines the cluster positions and the second one determines randomly the position of 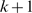 points within each cluster according to an arbitrary density probability function. The correspondance between both representations is straightforward and the power spectrum density can be computed analytically with the Neyman-Scott equation [23],[24],[51]

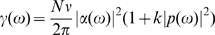
(5)where 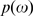 is the Fourier transform of the delay distribution, 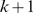 is the number of synchronous neurons and 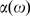 is the Fourier transform of the synaptic filtering. In Eq. 5, the factor 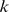 can also be written 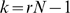 where 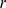 is the ratio of synchronous neurons which does not depend anymore on 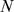.

In this paper, we are interested in the power-law frequency-scaling in the temporal power spectrum density (PSD). Eq. 5 relates the delay distribution to the PSD so that a power-law behaviour at the conductance level needs a power-law scaling in the delay distribution. Therefore, the delay associated with each synapse was randomly chosen from a distribution proportional to 
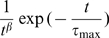
. The exponential term is added to avoid oscillations in the PSD due to an abrupt cut-off [6] with 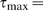
10 ms. The parameter 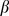 is varied over the simulations and modulates the spread of temporal correlations. The presynaptic neurons are synchronously active according to the parameter 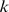. The output frequency-scaling exponent (to be defined below) measured in the PSD (Eq.5) is thus equal to 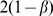.

### *In vitro* Preparation

*In vitro* experiments were performed on 350 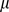
m-thick sagittal slices from the lateral portions of rat occipital cortex. Wistar Rats, 4–6 weeks old (CNRS, Gif-sur-Yvette), were anesthetized with sodium pentobarbital (30 mg/kg) before craniectomy and cortex removal. The slices were maintained in an interface style recording chamber at 34–35°C. Slices were prepared on a DSK microslicer (Ted Pella, Redding, CA) in a slice solution in which the NaCl was replaced with sucrose while maintaining an osmolarity of 314 mosM. During recording, the slices were incubated in slice solution containing (in mM) 126 NaCl, 2.5 KCl, 1.2 MgSO_4_, 1.25 NaHPO_4_, 2 CaCl_2_, 26 NaHCO_3_, and 25 dextrose and aerated with 95% O_2_-5% CO_2_ to a final pH of 7.4. After 30 minutes to 2 hours of recovery, intracellular recordings were performed in deep layers (layer IV–VI) in electrophysiologically identified regular spiking and intrinsically bursting cells. Micropipettes were filled with 1.2–2 M potassium acetate and 4 mM KCl and had resistances of 80–100 M after bevelling. The dynamic-clamp technique [53],[54] coupled with an Active Electrode Compensation (AEC) method that we developed and validated recently *in vivo* and *in vitro* [24] was used to inject computer-generated conductances in real neurons. The AEC method allows the removal in real time of electrode noise from intracellular voltage recordings. Dynamic-clamp experiments were run using the Real Time-NEURON environment [55], which is a modified version of NEURON 6.0 [49].

The dynamic-clamp protocol was used to insert the fluctuating conductances underlying synaptic noise in cortical neurons using the previous model, the post-synaptic neuron being now the recorded neuron, similar to a previous study [56]. The injected current is determined from the fluctuating excitatory and inhibitory conductances as well as from the difference of the membrane voltage from the respective reversal potentials.

### Power Spectrum Analysis

Spikes were removed from the original traces and replaced by a low-pass filtered version of the trace. To control the validity of this procedure, we compared whenever possible the power spectrum obtained from the interpolated trace with an identical trace generated without threshold. In all cases we observed that injecting a given conductance trace into a neuronal model and then removing the spikes gave the same power spectrum as injecting the same conductance in a neuronal model without spike threshold (Fig. S2). The spectra were computed with the multi-taper method [57], which allows a better estimation of the power-laws than the standard periodogram methods. Results were similar when using the Welch method and the Goertzel algorithm [58].

We then determined the frequency-scaling exponent by linear regression on a log-log representation of the PSD, for the range 75–200 Hz. Similar results were obtained for lower bounds above 50 Hz, and higher bounds below 200 Hz. Estimation of the scaling exponent from multifractal methods gave similar values. For the *in vitro* data, we also estimated the frequency-scaling exponent by fitting a generalized Lorentzian function [59], which gave equivalent relative values.

We chose to use the linear fit for its simplicity, and because it is easy to quantify the goodness of fit, and thus to assess the power-law scaling over the frequency band chosen. In comparison, the Lorentzian fit is very accurate when considering controlled models where the cut-off frequencies can be easily found or computed, but this model gave inaccurate results when applied to *in vivo* data because it can not account for the low frequency regime, which is strongly modulated by the stimulus. Finally, the multifractal analysis gave us no control over the goodness of fit. In the case of the recurrent network, the fit was performed between 75 and 200 Hz. Using narrower bands gives similar results. In the *in vitro* measurements, the absolute values of the frequency-scaling exponent displayed significant variations because of the available scaling region. Our study focused on the *modulation* of the frequency-scaling, rather than on absolute values, the relative values of the frequency-scaling exponent are shown for *in vitro* experiments and the corresponding models for each linear region of the PSD. For the model studies, unless otherwise mentioned, we systematically subtracted the value obtained for a classical Poisson input. For the *in vitro* study, the reference was the frequency-scaling exponent obtained with the input parameter 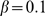, averaged over the different conditions tested. In this case, measuring the relative values also removed the cell-to-cell variability of the absolute values.

The total input conductance is reported to be about three times the leak conductance 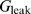 in the anaesthetized cat [26]. This is also what we used in our model and in the conductance injection *in vitro*. As a consequence, the cut-off frequency of the synaptic and membrane filtering are below the frequency band used for our fitting (they did not exceed 75 Hz), and could not affect our estimates (this point is futher discussed in the Results section).

### Multifractal Analysis

The multifractal analysis characterizes the scaling behavior of a signal 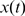 [60]. For each point 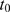, the Hölder exponent 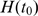 is defined as the maximal value 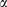 such that there exists a polynomial 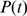, with 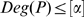, a positive constant 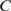, and an interval around 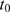 where for any 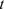


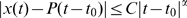
(6)This coefficient 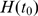 reflects the scaling behaviour around the point 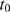. The singularity spectrum 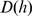 is the Haussdorf dimension of 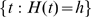. It thus describes how the singularities are distributed in the signal. A particular example is the self-similar process (also called monofractal), where 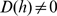 only at one point 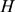, where 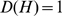. The practical estimation of the singularity spectrum is made difficult by the finite size of the signal, and by its discrete nature. However, the wavelet formalism allows a robust estimation of 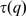, which is the Legendre transform of the singularity spectrum:
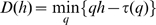
(7)In the case of a monofractal/scale-invariant process, 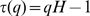, H being its unique Hölder exponent. This corresponds to a fractional Brownian process. Note that H is related to the PSD slope which is equal to 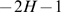. The curvature of 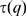 quantifies the deviation from monofractality. The slope and the curvature are respectively the first and second moments of the singularity spectrum. We used an algorithm based on wavelet leaders [20],[61] which directly estimates these two values.

### Fano Factor and Power-Law in the Spiking Activity

Fano factors and power-laws on these Fano factors were measured as in [6]. To compute the Fano Factor for a given time bin, we counted the number of spikes in each time bin and took the ratio of the spike-count variance to the mean spike-count. The power-law was estimated by computing this Fano Factor over a large range of time bins. This function was then represented in a log-log scale, and the slope of the curve was estimated by linear regression. This gives the frequency-scaling exponent of the spiking activity through the Fano Factor 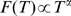 where 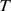 is the time bin and 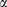 the scaling exponent.

## Supporting Information

Figure S1 Effect of heterogeneous synaptic weights and synaptic waveform on the power law frequency scaling exponent. (A–B) V_m_ frequency-scaling exponent changes for different input frequencies ν and for heterogeneous synaptic strengths. The synaptic strengths are randomly distributed for each incoming synaptic spike train according to a Gaussian distribution whose standard deviation is half the mean value in this case. These controls were performed with integrate-and-fire neurons (panel A) and Hodgkin-Huxley neurons (panel B). The synchrony percentage was kept a 6% and there was no correlation between excitatory and inhibitory synaptic inputs. Error bars are the standard deviation over the trials. The bold line represents the average across cells and trials. (C–D) Variation of the value of the frequency-scaling exponent at the membrane potential level for excitatory input only as a function of the parameters β_exc_ and for β-synapses (r = 3%). (C) Illustration of the PSD modulation on a log-log scale for different values of the parameter β_exc_ ranging from 0 (light blue) to 1 (dark blue). In the inset, a stereotypic synaptic time course is represented (with a time rise of 1 ms). (D) Variation of the output frequency-scaling exponent with the β_exc_ parameter.
(0.41 MB EPS)

Figure S2 Illustration of the spike filtering algorithm for neuron models with and without spiking mechanism. (A) Injection of correlated synaptic input to a HH model. Blue: raw trace; Red: after spike filtering. (B) Power spectra density corresponding to panel A. (C) injection of the same synaptic input in a COBA model without threshold (green), superimposed to the HH-spike-filtered trace plotted in panel A. (D) Power spectra density of the the two traces displayed in panel C: COBA without threshold and HH with spike filtered.
(0.97 MB EPS)

Figure S3 Influence of the different integrative time constants on the PSD frequency scaling. (A) V_m_ power spectra for different levels of correlation in the input (blue: Poisson input; red: correlated input with k = 6% and β = 0). The level of conductance is low in this condition (G_tot_ = 0.23G_leak_). The dotted coloured lines indicate the linear fits over the high frequency region delimited by the vertical dashed gray line. (B) same PSD, but for a very high conductance state (G_tot_ = 12G_leak_). The four fits correspond to fit in different frequency bands, for the two PSDs. To illustrate more precisely the differential effect of the conductance state and of the input correlations on the frequency-scaling exponent, we show several examples of V_m_ power spectra for two different levels of global conductance regime, and two different β_inh_ = β_exc_ parameters. In the low conductance state (panel A), the power spectrum is composed of two linear regions separated by a unique cut-off, which is determined by the time constants of the synaptic and membrane filtering. In the very high conductance state (panel B), these two time constants are clearly different, so the power spectrum shows three linear regions separated by two cut-offs. Very large (and surely not plausible in biological conditions) changes of the conductance state thus displaced the second frequency cut-off, but still did not affect the relative slope in the linear regions. Decreasing the β parameter increases the slope over both frequency bands and relative changes of the frequency-scaling exponent have the same magnitude in these different regions. This shows that the relative modulation observed is not dependent on the specific frequency band chosen to estimate the PSD slope, since it can be observed over a large range of frequencies. Furthermore, this figure illustrates the differential effect of the conductance state, and of the correlation state, on the power spectrum. Opposite to the latter, the former does no affect the scaling exponent.
(0.52 MB EPS)

Table S1 Frequency-scaling exponents for detailed neuron models. Neuron models were obtained from neuronal morphologies reconstructed from a layer III cell (upper table) and a layer VI cell (lower table) of the cat cerebral cortex (see methods). The frequency-scaling exponent is computed for different synaptic input firing rates and different levels of synchrony. Three levels of incoming synaptic activity have been considered, following (Destexhe & Paré, 1999) : a high-conductance state (HC) with ν_exc_ = 1 Hz, ν_inh_ = 5.5 Hz; a low-conductance state (LC) with ν_exc_ = ν_inh_ = 0.5 Hz and a very low-conductance state (VLC) with ν_exc_ = ν_inh_ = 0.1 Hz. Each condition was performed with two levels of synchrony between synaptic spike trains, r = 0% and r = 1.5% respectively. Frequency-scaling exponents barely changed with increasing firing rate for both uncorrelated and correlated inputs, for both cells. However, the frequency-scaling exponent was affected by the level of synchrony, as expected from our previous results. These simulations show that the relative modulations of the scaling exponent are mostly due to correlation changes, while conductance changes have a negligible effect.
(0.01 MB PDF)
