# Supplementary material for: Network-State Modulation of Power-Law Frequency-Scaling in Visual Cortical Neurons
Source: PLoS Comput Biol. 2009 Sep 25;5(9):e1000519. doi: 10.1371/journal.pcbi.1000519 (PMC2740863; doi:10.1371/journal.pcbi.1000519)
Supplement: Table S1 — Frequency-scaling exponents for detailed neuron models. Neuron models were obtained from neuronal morphologies reconstructed from a layer III cell (upper table) and a layer VI cell (lower table) of the cat cerebral cortex (see methods). The frequency-scaling exponent is computed for different synaptic input firing rates and different levels of synchrony. Three levels of incoming synaptic activity have been considered, following (Destexhe & Paré, 1999) : a high-conductance state (HC) with νexc = 1 Hz, νinh = 5.5 Hz; a low-conductance state (LC) with νexc = νinh = 0.5 Hz and a very low-conductance state (VLC) with νexc = νinh = 0.1 Hz. Each condition was performed with two levels of synchrony between synaptic spike trains, r = 0% and r = 1.5% respectively. Frequency-scaling exponents barely changed with increasing firing rate for both uncorrelated and correlated inputs, for both cells. However, the frequency-scaling exponent was affected by the level of synchrony, as expected from our previous results. These simulations show that the relative modulations of the scaling exponent are mostly due to correlation changes, while conductance changes have a negligible effect. (0.01 MB PDF) [file pcbi.1000519.s004.pdf]

Layer III cell	HC	LC	VLC
r = 0%	3.40	3.39	3.38
r = 1.5%	3.56	3.62	3.63

Layer VI cell	HC	LC	VLC
r = 0%	3.39	3.36	3.29
r = 1.5%	3.54	3.55	3.51
